# Simulated climate effects of desert irrigation geoengineering

**DOI:** 10.1038/srep46443

**Published:** 2017-04-18

**Authors:** Wei Cheng, John C. Moore, Long Cao, Duoying Ji, Liyun Zhao

**Affiliations:** 1 Joint Center for Global Change Studies, College of Global Change and Earth System Science, Beijing Normal University, Beijing 100875, China; 2 School of Earth Science, Zhejiang University, Hangzhou, Zhejiang 310027, China

## Abstract

Geoengineering, the deliberate large-scale manipulation of earth’s energy balance to counteract global warming, is an attractive proposition for sparsely populated deserts. We use the BNU and UVic Earth system models to simulate the effects of irrigating deserts under the RCP8.5 scenario. Previous studies focused on increasing desert albedo to reduce global warming; in contrast we examine how extending afforestation and ecological projects, that successfully improve regional environments, fair for geoengineering purposes. As expected desert irrigation allows vegetation to grow, with bare soil or grass gradually becoming shrub or tree covered, with increases in terrestrial carbon storage of 90.3 Pg C (UVic-ESCM) – 143.9 Pg C (BNU-ESM). Irrigating global deserts makes the land surface temperature decrease by 0.48 °C and land precipitation increase by 100 mm yr^−1^. In the irrigated areas, BNU-ESM simulates significant cooling of up to 4.2 °C owing to the increases in low cloud and latent heat which counteract the warming effect due to decreased surface albedo. Large volumes of water would be required to maintain global desert irrigation, equivalent 10 mm/year of global sea level (BNU-ESM) compensate for evapotranspiration losses. Differences in climate responses between the deserts prompt research into tailored albedo-irrigation schemes.

Desertification over the past several decades has provided a significant cooling effect on the earth’s surface, equivalent to about 20% of the warming due to global anthropogenic CO_2_ emissions over the same period^1^. This strong climatic effect promoted suggestions for increasing desert albedo e.g., by covering it with reflective material, as a potential geoengineering scheme^2^. However, simulations suggest that increasing desert albedo or expanding deserts^3^ would cause significant changes in global-scale circulation, the hydrological cycle, regional-scale precipitation patterns, and a large reduction in the intensity of the Indian and African monsoons^4^. In addition, desert expansion would also lead to the loss of productive land and the modification of natural arid ecosystems^5^.

Afforestation, the conversion of croplands or marginal lands into forests, is in some locations such as China, a cost-effective way to sequester CO_2_ from the atmosphere and an environmentally friendly way to ameliorate sand storms and mitigate desert encroachment^6^. The United Nations considers afforestation to be a key governmental climate-change mitigation strategy^7^. Afforestation not only contributes to increased carbon storage^8^ but also alters local albedo and turbulent energy fluxes, and enhances evapotranspiration that feedbacks on local and regional climate^9^,^10^. An expansion of relatively dark forest area increases the absorption of solar energy and increases surface temperature, particularly in regions where the land surface is unable to compensate with latent heat flux due to water limitation^11^. Afforestation can therefore result in net climate warming, particularly at high latitudes^7^. However, in the tropics, afforestation tends to have a net cooling effect because increased evaporative cooling overwhelms the heating effect of lowered surface albedo^12^.

Large increases in plant cover at geoengineering scales would require the addition of water to arid regions where they cannot grow at present. The climatic impact of irrigation in agriculture and/or desert regions has been variously assessed previously^8^,^9^,^13^,^14^,^15^,^16^. Agricultural irrigation can produce significant regional cooling with strong seasonal variability^13^,^14^ and regional increases in precipitation^14^, but has a negligible effect on global average near-surface temperatures^15^,^16^. There have been few desert irrigation or afforestation simulation studies^8^,^9^. Ornstein *et al*.^9^ used GISS ModelE General Circulation Model to simulate Saharan and Australian deserts irrigation with prescribed vegetation and phenology. Keller *et al*.^8^ used the University of Victoria Earth System Climate Model (UVic-ESCM) to study climate effects and carbon uptakes of afforestation geoengineering in the deserts of North Africa and Australia.

In this study, we use the BNU-ESM and the intermediate complexity UVic-ESCM Earth System Models to conduct idealized deserts irrigation geoengineering simulations during the 21^st^ century. We conducted five sets of simulation experiments: no irrigation as a baseline (*GE_none*), irrigation of global deserts (*GE_Globe*), irrigation of Northwest China deserts (*GE_China*), irrigation of Australian deserts (*GE_Australia*), and irrigation of North African deserts (*GE_Africa*) (Supplementary Table S1). In each scenario we simulate irrigation by forcing soil moisture to be constant at 360 kg m^−2^ for UVic (following Keller *et al*.^8^) and by daily restoring the moisture content in the 3.433 m deep BNU-ESM soil column to be 800 mm thick, so as to avoid excessive runoff into ocean from the irrigated regions. All simulations are under the Intergovernmental Panel on Climate Change (IPCC) representative concentration pathway, RCP8.5^17^ CO_2_ concentration scenario, which can only take the biogeophysical effects from irrigation, but exclude biogeochemical responses. We perform these simulations to illustrate the likely response of carbon reservoirs and climate to irrigating global and individual deserts geoengineering during the 21^st^ century. The UVic-ESCM uses a reduced complexity atmosphere model with no parametrization for clouds and their feedbacks, nor atmospheric transport dynamic processes^18^. Therefore, the UVic-simulated climate effect of irrigation might be less reliable than that of BNU-ESM, and for results from UVic-ESCM simulations, we focus on the effect of irrigation on carbon reservoirs and land surface cover. A detailed description of the models used and simulation experiments is provided in the Method section.

## Results

### Response of terrestrial carbon cycle to irrigation

In the each of the irrigated regions, desert irrigation lead to decreases in the area of bare soil and C_4_ grasses (Supplementary Figs S1 and S2) which are less adapted to wetter environments^19^. At the same time, the fractional area of C_3_ grasses increases initially, but then declines as shrubs take over (Supplementary Fig. S2). Eventually irrigation increases the fractional area of broadleaf trees (Supplementary Figs S1 and S2). By the end of simulation, the modeled predominant plant functional type in the irrigated areas is broadleaf tress for BNU-ESM and shrubs for UVic-ESCM, owing to the different vegetation dynamics used: the Lund–Potsdam–Jena (LPJ) model^20^ for BNU-ESM; and the Lotka-Volterra approach^21^ in UVic-ESCM. However, with BNU-ESM, irrigation could not make vegetation grow in Australia, likely due to simulated limitation in soil organic matter content. These changes in vegetation cover have a remarkable effect on terrestrial carbon cycle.

**Figure 1:**
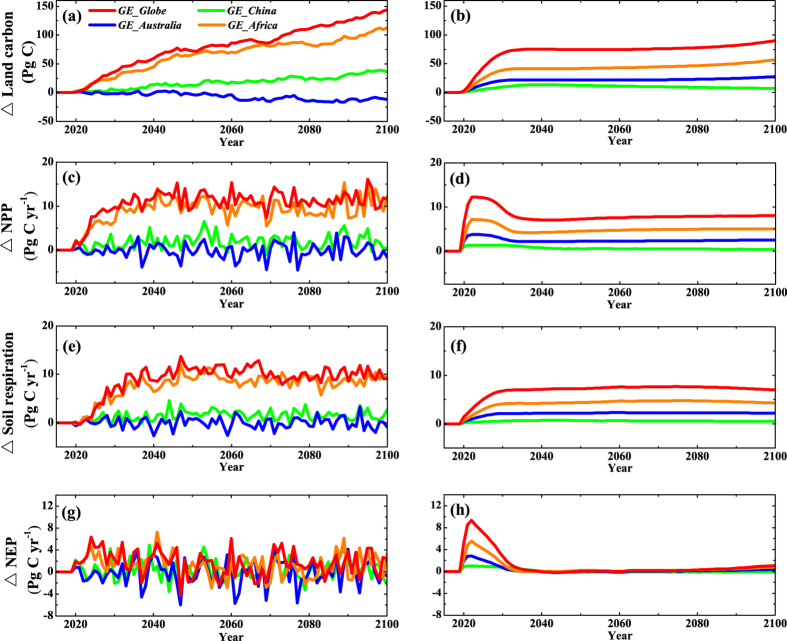
Simulated temporal evolution of carbon changes anomalies, relative to *GE_none,* due to irrigation desert geoengineering. BNU-ESM results are shown in the left column, and UVic-ESCM results are shown in the right column. (**a**,**b**) global total land carbon; (**c**,**d**) global net primary productivity (NPP); (**e**,**f**) global soil respiration (RES); (**g**,**h**) net ecosystem productivity (NEP, NPP minus RES). Desert irrigation starts from 2020 to 2100. Values are annual and global means. This figure was plotted using Origin version 8.5 from OriginLab.

Increased shrub and tree cover in the irrigated areas increases both vegetation and soil terrestrial carbon reservoirs. By the end of simulation, BNU-ESM suggests increases in global vegetation and soil carbon caused by global desert irrigation of 73.1 and 70.8 Pg C respectively (Supplementary Fig. S3a,c). For UVic-ESCM, corresponding increases are 45.4 and 44.9 Pg C (Supplementary Fig. S3b,d). The greatest simulated increase is in vegetation carbon, reflecting the delay between production of additional living biomass and transfer to the litter and soil pools. *GE_Globe* increases total terrestrial carbon by 143.9 Pg C (7.6%) in BNU-ESM and 90.3 Pg C (4%) in UVic-ESCM compared with *GE_none* (Fig. 1a,b), and the increase in terrestrial carbon under *GE_China, GE_Australia* and *GE_Africa* are 7.5% (6.8 Pg C), 30.3% (27.3 Pg C) and 62.7% (56.6 Pg C) of *GE_Globe* using UVic-ESCM. These models predict increases in global net primary productivity (NPP), soil respiration (RES) and net ecosystem productivity (NEP, NPP minus RES) of 10.9, 9.2 and 1.7 Pg C yr^−1^ individually for BNU-ESM and 8.1, 7 and 1.1 Pg C yr^−1^ individually for UVic-ESCM with *GE_Globe* compared with *GE_none* averaged over the whole simulations. The increase of NPP in the irrigated area is due to vegetation growth, however, the increase of RES is affected by the increase of soil moisture, and the accumulation of litter and organic matter in the irrigated area. During 2020 to 2030, the average NEP anomaly under *GE_Globe* scenarios compared with *GE_none* is about 3.3 Pg C yr^−1^ for BNU-ESM and 6.3 Pg C yr^−1^ for UVic-ESCM.

**Figure 2:**
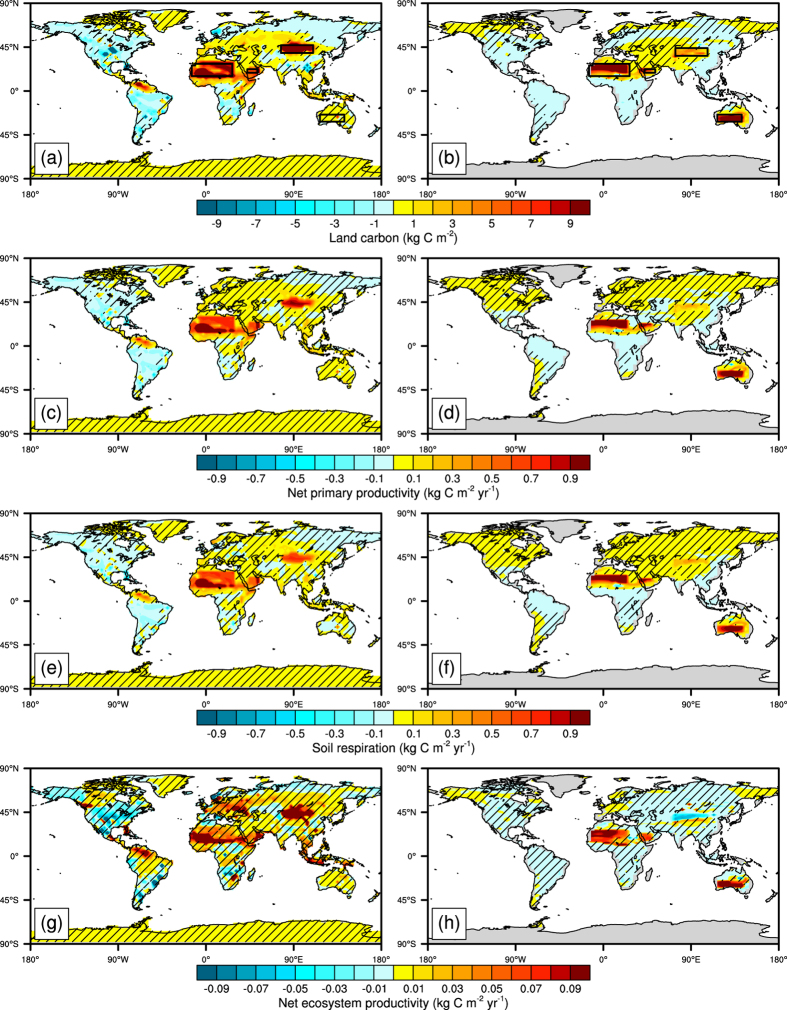
Maps showing model-simulated land carbon changes for *GE_Globe* compared with *GE_none* for 2071–2100. BNU-ESM results are shown in the left column, and UVic-ESCM results are shown in the right column. (**a**,**b**) total land carbon; (**e**,**f)** net primary productivity (NPP); (**g**,**h**) soil respiration (RES); (**c**,**d**) net ecosystem productivity (NEP, NPP minus RES). The boxed regions in (**a**) and (**b**) show where irrigation was applied. Hatched areas are regions where changes are not statistically significant at the 5% level using the Student’s t-test. The Student’s t-test and maps were produced using NCAR Command Language (NCL)^60^ version 6.1.2 (http://www.ncl.ucar.edu/).

In the North African irrigated areas, the simulated land carbon rises to more than 7.1 and 10.2 kg C m^−2^, and NEP (NPP minus RES) to 0.065 and 0.095 kg C m^−2^ yr^−1^ (Fig. 2a,b,g,h) during 2071 to 2100 for UVic-ESCM and BNU-ESM, individually, because of the different vegetation dynamics between the two models we used (Supplementary Figs S1c and S2c). However, in Australian irrigated areas, there are no increases in land carbon and NEP using BNU-ESM (Fig. 2a,g) as irrigation fails to make vegetation grow (Supplementary Fig. S1c). In the Northwest China irrigated areas UVic-ESCM simulated NEP anomaly is about −0.031 kg C m^−2^ yr^−1^ over the same period ((Fig. 2h) as the carbon uptake from shrubs is offset by decreases in C_3_ grasses (Supplementary Fig. S2b).

### Response of surface albedo to irrigation

**Figure 3:**
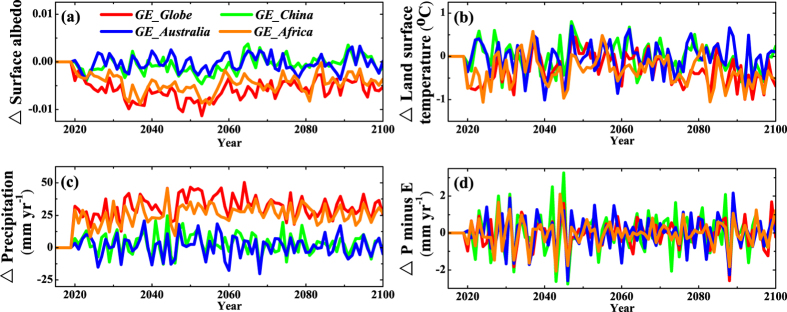
BNU-ESM simulated temporal evolution of climate changes anomalies, relative to *GE_none,* due to irrigation desert geoengineering. (**a**) global average surface albedo; (**b**) global average land surface air temperature; (**c**) global average precipitation and (**d**) global average precipitation minus evapotranspiration (P-E). Desert irrigation starts from 2020 to 2100. Values are annual global means. This figure was plotted using Origin version 8.5 from OriginLab.

**Figure 4:**
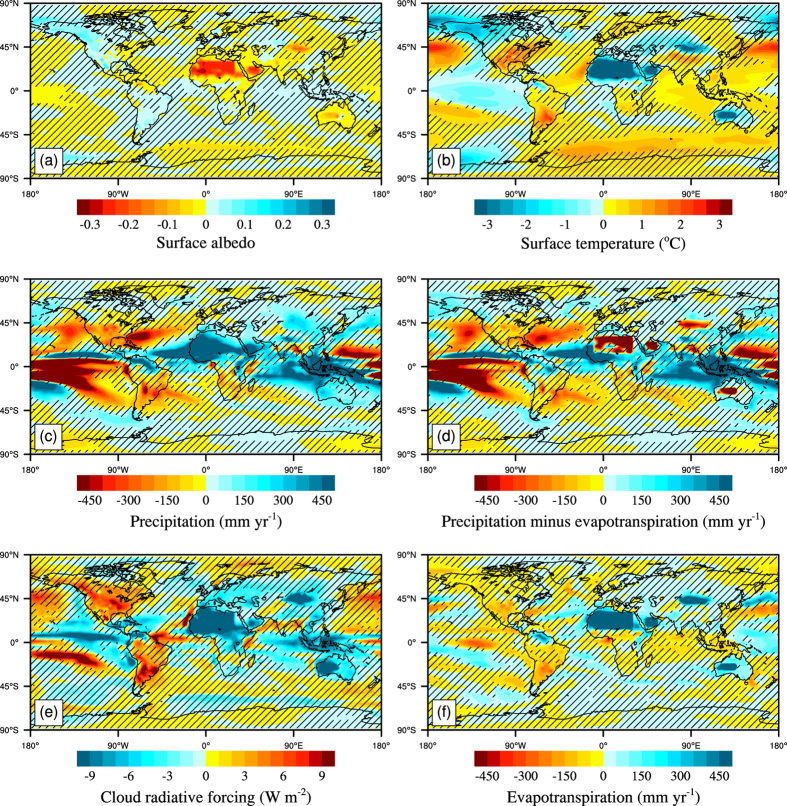
Maps showing BNU-ESM simulated climate impacts for *GE_Globe* compared with *GE_none* for 2071–2100. (**a**) surface albedo, (**b**) surface temperature, (**c**) precipitation, (**d**) precipitation minus evapotranspiration (P-E), (**e**) TOA cloud net radiative forcing (longwave and shortwave) and (**f**) evapotranspiration. Hatched areas are regions where changes are not statistically significant at the 5% level using the Student’s t-test. The Student’s t-test and maps were produced using NCAR Command Language (NCL)^60^ version 6.1.2 (http://www.ncl.ucar.edu/).

The models’ terrestrial carbon cycle modules determine the exchange of CO_2_ between the land and the atmosphere. They are coupled to the physical climate through changes in vegetation growth (Supplementary Fig. S1), vegetation distribution and leaf area index, which affect the surface albedo, the evapotranspiration flux and so on. Relative to the *GE_none* simulation, BNU-ESM simulated global mean surface albedo under *GE_Globe* and *GE_Africa* abruptly decrease in the first 7 years (Fig. 3a) as the desert is covered with vegetation that has a lower albedo, particularly in the North African irrigated areas (Fig. 4a), then albedo continues to gradually decrease with inter-annual variability until 2053 (Fig. 3a). In western North America increased snow cover (Supplementary Fig. S4) leads to an albedo increase of over 0.1 under global desert irrigation scenario compared with the *GE_none* during 2071–2100 (Fig. 4a). The snow increases are due to greater moisture supply from the northern Pacific driven by sea level pressure rises there (Supplementary Figs S5 and S6). Subsequent positive albedo feedback cools the surface locally. In contrast, under *GE_China* simulation albedo changes are small because the bare soil fraction in Northwest China is much less before irrigation starts than in North Africa (Supplementary Fig. 1b,d; Table 1). Under *GE_Australia* simulation, no increased vegetation occurs, however, the increase in the soil moisture leads to the small decrease of albedo locally (Fig. 4a). During 2071 to 2100, the average surface albedo changes in irrigated areas and non-irrigated area on land under *GE_Globe* scenarios compared with *GE_none* are −0.152 and −0.004 (Supplementary Fig. S7a; Table 1), with the local responses to irrigation dominating changes in remote regions.

### Response of surface temperature to irrigation

In our simulations, irrigation affects surface temperature mainly through its effect on surface albedo, latent heat and cloud effect. Decreases in surface albedo associated with vegetation growth due to irrigation act to warm the surface, but the increases in evapotranspiration and low cloud cover due to irrigation and vegetation growth also cool the surface. Compared to CO_2_ induced warming, irrigation has a tiny effect on surface temperatures. Simulated global mean land surface temperature with global desert irrigation for the period 2020 and 2100 cools by 0.31 °C (Fig. 3b), which is, of course, much less than the 4.8 °C rise in global mean land temperature caused by increasing atmospheric CO_2_ concentrations under the *GE_none* scenario driven by RCP8.5 concentrations. However, the global mean surface temperature caused by global desert irrigation warms by 0.08 °C due to a rise of 0.24 °C in mean ocean temperature (Supplementary Fig. S8b,d). Under the *GE_Globe* and *GE_Africa* scenarios, latent heat flux abruptly increases in the first 5 years due to the increase of evapotranspiration in irrigated areas (Supplementary Fig. S7d), producing a net global land cooling of 1 °C (Fig. 3b).

Averaged over the 2071 to 2100 period, *GE_Globe* and *GE_Africa* produce global land average temperature anomalies of about −0.48 and −0.46 °C respectively relative to *GE_none* (Fig. 3b). However, global mean land temperature anomalies are near zero (Fig. 3b and Supplementary Table S2) under the *GE_China* and *GE_Australia* for the same period. The cooling effects are concentrated in the irrigated and adjacent areas (Fig. 4b), where decrease of up to −4.2 °C (Supplementary Fig. S7b). The cooling effect is due to TOA cloud net radiative forcing (longwave and shortwave) caused by the increases in low cloud cover fraction (Fig. 4e and Supplementary Fig. S9) and latent heat flux due to increases in evapotranspiration (Fig. 4f), which overwhelm the warming from decreased surface albedo (Fig. 4a). Modeled surface air temperatures cool over western North America by up to 2 °C due to increased snow cover (Fig. 4a and Supplementary Fig. S4). Over ocean, the temperature changes are consistent with cloud fraction, TOA cloud net radiative forcing and sea surface albedo changes. For example the positive temperature anomaly over the north Pacific is linked to positive TOA net cloud radiative forcing and decreased low cloud fraction (Fig. 4e and Supplementary Fig. S9), and the small positive temperature anomaly over the Southern Ocean to albedo changes probably caused by sea ice loss and changes in Antarctic Circumpolar Current (ACC) (Fig. 4a and Supplementary Fig. S10a,b).

### Response of precipitation and evapotranspiration to irrigation

As a result of increased evapotranspiration associated with vegetation growth, desert irrigation increases global average precipitation, especially in and around the irrigated areas. Compared with *GE_none* in 2071 to 2100, increases in global mean precipitation are about 30.4, 2.3, 1.0 and 26.5 mm yr^−1^ for *GE_Globe, GE_China, GE_Australia* and *GE_Africa* respectively (Fig. 3c). Over land areas precipitation increases by up to 99.6 mm yr^−1^ (Supplementary Fig. S7c), but by only 1.7 mm yr^−1^ over the oceans during the 2071 to 2100 period (Supplementary Fig. S8h). Positive precipitation anomalies equator-ward and westward of the irrigation in North Africa and Australia, and westward of the irrigation in Northwest China (Fig. 4c) are driven by climatological winds, geostrophic adjustment and diffusion carrying moisture from the irrigated regions (Supplementary Fig. S5). Increases in tropical easterlies is consistent with enhancement of the Walker circulation due to increased sea-level pressure differences between eastern Pacific Ocean (130–80° W, 10° S–10° N) and Indonesia (110–160° E, 10° S–10° N) (Supplementary Figs S6 and S11), and in-line with model simulations under greenhouse gas forcing^22^ and recent observations^23^.

Evapotranspiration increases in irrigated areas because of increases in both vegetation cover and soil moisture (Fig. 4f). Global mean precipitation minus evapotranspiration (P-E) (Fig. 3d) decreases for all irrigation cases (Table 1) relative to *GE_none.* Averaged over years 2071–2100 regional irrigation in North Africa, Australia Northwest China causes decreases in P-E of up to −276.2 mm yr^−1^ (Fig. 4d, Supplementary Fig. S7d). For the same period, P-E over land increases by 4.5 mm yr^−1^ while it decreases by 1.8 mm yr^−1^ over oceans relative to *GE_none* (Supplementary Fig. S7d).

## Discussion

We may validate our simulations by comparison with some previous published work on the regional climatic impacts of irrigation^13^,^14^,^15^,^16^; and with irrigation impacts on carbon storage^6^,^24^,^25^. The models we use have also been thoroughly validated against observations (see Methods), and via model intercomparisons of future greenhouse and geoengineered climates^26^,^27^,^28^. Kueppers *et al*.^13^ used a regional climate model (RegCM3) to deduce that a local annual mean cooling of 1.6 °C (1981–2000) was caused by irrigated agriculture in California, which may be compared with the BNU-ESM simulated local cooling of 4.2 °C in desert irrigated areas. This cooling is more comparable with atmospheric general circulation model (GISS ModelE) simulations of 20th century irrigation^14^, which suggested peak regional cooling of about 3 °C in the northwestern portions of the Indian subcontinent. While global agricultural irrigation has altered climate significantly in some regions during the late 20^th^ century, there has been a negligible effect on average near-surface temperatures^14^,^15^,^16^, in agreement with our very small (0.08 °C) mean temperature difference simulated by desert irrigation over 2071–2100. Our simulated changes in carbon stocks are comparable with field measurements in deserts. The UVic-ESCM and BNU-ESM simulated average NEP (NPP minus RES) in the North African irrigated areas rises to 0.065 and 0.095 kg C m^−2^ yr^−1^ (Fig. 2a,b,g,h), which are both consistent with net uptake of carbon ranging from 0.033 to 0.127 kg C m^−2^ yr^−1^ in field experiments in the Tengger Desert by Yang *et al*.^6^, northern Chihuahuan Desert by Petrie *et al*.^24^ and Mojave Desert by Jasoni *et al*.^25^.

There are only a few existing studies on climate effect of desert irrigation or afforestation^8^,^9^. Ornstein *et al*.^9^, using the GISS ModelE coupled climate model, simulated the effect of afforestation by replacing the deserts of Sahara and Australia with evergreen tropical rain forest and changing the corresponding desert soil to a composition typical for rain forest. They found that afforestation has very localized effects with temperature decreases of 2 to 3 °C and precipitation increases of 2 to 3 mm day^−1^ over North African and Australian deserts. In contrast, Keller *et al*.^8^, using the UVic-ESCM, simulated the climate effect of afforestation by forcing the soil moisture to be 360 kg m^−2^ in the deserts of North Africa and the Australia. They found that the albedo change caused by simulated afforestation resulted in a global mean temperature warming of 0.1 °C and an 18 mm yr^−1^ increase in precipitation in 2100 relative to no climate engineering. We, using the BNU-ESM, find that irrigating global and North African deserts produces significant cooling in the irrigated and adjacent areas, where decreases of up to −4.2 °C occur by 2071–2100, mainly as a result of increases in low cloud cover and latent heat flux, which counteracts the warming effect associated with reduced surface albedo. However, there is a warming of 0.08 °C in global mean surface temperature, which the cooling of 0.31 °C in global mean land surface temperature is overwhelmed by the warming of 0.24 °C in global mean ocean surface temperature, from irrigation of global deserts during the simulations. Global average precipitation by 2071–2100 under *GE_Globe* increases by 30.4 mm yr^−1^ (Fig. 3j) compared with *GE_none*. The increases in precipitation under *GE_China, GE_Australia* and *GE_Africa* are 7.6% (2.3 mm yr^−1^), 3.3% (1.0 mm yr^−1^) and 87.2% (26.5 mm yr^−1^) of *GE_Globe*. Puma *et al*.^14^ and Sacks *et al*.^15^ estimated that agriculture irrigation increased global land average precipitation by 9.5–4.3 mm yr^−1^, during the late 20th century, both of which are much lower than the increase in precipitation under *GE_Globe*, but this may be explained by the different irrigation rates^16^. Evapotranspiration is high in the irrigated areas because of increases in both vegetation cover and soil moisture, hence average precipitation minus evapotranspiration (P-E) anomalies in the irrigated area are negative (Fig. 4d and Table 1). In contrast, Ornstein *et al*.^9^ simulations produced considerable extra rainfall over irrigated new forests, almost offsetting the 2 to 3 mm day^−1^ increases in evaporation. GISS ModelE as used by Ornstein *et al*.^9^ included a rather simple vegetation module compared with that in the BNU-ESM^29^.

In our simulations using the BNU-ESM and UVic-ESCM model we rely on natural vegetation succession, though actually a more likely scenario would be to irrigate and plant. In practice irrigation would be done with a blend of drought-resistant trees and shrub species which use water efficiently and can tolerate the arid environment^30^. In our simulations, global desert irrigation increased the terrestrial carbon uptake by 6.4 Pg C yr^−1^ by the fifth year of irrigation for BNU-ESM and by 9.4 Pg C yr^−1^ in only the third year of irrigation for UVic-ESCM, which has a faster vegetation succession than the BNU-ESM. *GE_China, GE_Australia* and *GE_Africa* peak sequestration rates are 34% (11%), 14% (30%) and 72% (60%) of *GE_Globe* using BNU-ESM (UVic-ESCM) (Fig. 1g,h). Keller *et al*.^8^ finds a similar global peak removal rate, while Ornstein *et al*.^9^ estimated the terrestrial carbon reservoir flux from desert afforestation at about 8 Pg C yr^−1^ in the first 10 years. These rates are more than the carbon emission gap between Paris declaration of INDCs (Intended Nationally Determined Contributions) scenarios and emissions needed to meet a 2 °C peak warming^31^, which are about 2 Pg C yr^−1^.

Desert irrigation geoengineering needs huge amounts of fresh water. A first year rise in soil water content to daily restoring the moisture content in the 3.433 m deep BNU-ESM soil column to be 800 mm thick across the global deserts based on present-day soil water contents is equivalent to 7.6 mm of global mean sea level (2722 billion tons) (Supplementary Fig. S12f and Table 1). Ornstein *et al*.^9^ estimated an initial requirement of 4900 billion tons fresh water per year for Sahara desert irrigation (which they prescribe as requiring 500 mm yr^−1^ of precipitation), though in their simulation, this becomes self-sustaining due to increased rainfall over newly forested deserts. The required initial amount of fresh water estimated by Ornstein *et al*.^9^ is about twice our initial irrigation requirement, due to the different methods of irrigation or afforestation. More significantly in our simulations, global desert irrigation would require at least 3766 billion tons (10 mm of global sea level) per year to supplement net water lost by evapotranspiration (Fig. 4d and Supplementary Fig. S12h).

Keller *et al*.^8^ estimated that the higher soil moisture and precipitation caused by irrigation would raise the global mean sea level by about 13 cm by 2100 unless the water used for irrigation is desalinated seawater. However, economical irrigation limits water supply to maintain under-saturated soil, preventing runoff of excess water. The water required may be desalinated from sea water using reverse osmosis or permeable membrane desalination or extracted from deep aquifers^9^. This process would involve additional energy cost which will bring additional carbon emissions. Runoff and rainfall from desert irrigation would also regionally increase freshwater input to oceans thereby reducing coastal salinity. Lowered salinity ocean water in the upper ocean of the North Atlantic (Supplementary Fig. S13) under *GE_Globe* and *GE_Africa* may weaken Atlantic Meridional Overturning Circulation (AMOC)^32^ (Supplementary Fig. S10, Table S2).

Uncertainty in irrigation results appear quite large given the differences between our BNU-ESM results and those from the GISS ModelE, while UVic-ESCM seems reliable for carbon budgets, the lack of realistic atmospheric processes limits its usefulness in predicting regional climatic effects. The vegetation model exerts a large impact on the carbon budget. Furthermore, our simulations were driven by CO_2_ concentrations, and it only takes into account the biogeophysical effects from irrigation, but excludes biogeochemical responses. The increase of vegetation by irrigation or afforestation in coastal areas leads to increased sea-surface temperature owing to greater roughness and weaker winds over the adjacent coastal ocean, and then greater humidity, which is transported into the monsoon region by anomalous winds^33^. Furthermore, mineral dust aerosols affect atmosphere radiation budget by scattering and partly absorbing shortwave and longwave radiation^34^,^35^. Increasing soil moisture by irrigation and promoting vegetation growth means that less mineral dust aerosols will be mobilized and transported in the atmosphere^36^,^37^,^38^. Dust also has important fertilization effects in the ocean, and reductions may then lead to changes in ocean carbon budget. Last but not least, vegetation growth by irrigation or afforestation may induce more frequent occurrences of extreme rainfall events (flooding) over the coastal region and more frequent occurrence of heat waves and droughts over the semi-arid region^39^.

For these desert irrigation simulations, the changes in latent heat flux and low cloud are two dominant factors causing cooling (Fig. 4e,f). So we raise the idea that a combination of locally balancing artificial increases in albedo with irrigation driven increases in evapotranspiration may have much better prospects as a tool for desert climate engineering. This could be realized for example, by having both irrigated and highly reflective areas in the desert regions. An investigation of optimal desert geoengineering requires additional simulations in the future with more sophisticated models that contain high-resolution land cover data for reducing modeling uncertainties^40^.

## Methods

### Model description

The Beijing Normal University—Earth System Model (BNU-ESM)^29^ is a fully coupled Earth system model comprising elements representing the atmosphere (NCAR-CAM3.5), and land surface (BNU-CoLM3) at a T42 horizontal spectral resolution (approximately 2.81° × 2.81° transform grid), ocean (GFDL-MOM4p1) and sea-ice (LANL-CICE4.1) at a nominal latitude-longitude resolution of 1° (down to 1/3° within 10^o^ of the equatorial tropics) with 360 longitudinal grids and 200 latitudinal grids; and a coupler (improved NCAR-CPL6). It has an interactive carbon cycle model in the land component (BNU-DGVM (C/N)) based on LPJ and an ecosystem-biogeochemical module in the ocean component (IBGC). It has participated in the Coupled Model Intercomparison Project Phase 5 (CMIP5)^26^ and Geoengineering Model Intercomparison Project (GeoMIP)^27^.

As a member of CMIP5, BNU-ESM is based on several widely evaluated climate model components^41^,^42^,^43^,^44^. Ji *et al*.^29^ have validated the BNU-ESM’s performance in terms of the mean model state and the internal variability by comparisons between CMIP5 *piControl* and *historical* simulations and the observed datasets. BNU-ESM can in general, simulate observed features of the earth climate system as well as any other Earth System Model, including the climatological annual cycle of surface-air temperature and precipitation, annual cycle of cloud fraction and forcing, terrestrial gross primary productions (GPP) and soil organic carbon stocks (see Figs 4–9, refs 23 and 24 in Ji *et al*.^29^). BNU-ESM has proven to be a useful modeling tool and is being actively used by many researchers in prognostic simulations for both anthropogenic and geoengineering forcing scenarios^45^,^46^,^47^,^48^,^49^,^50^,^51^.

The University of Victoria Earth System Climate Model (UVic-ESCM) is a coupled climate-carbon cycle model with a horizontal resolution of 3.6° longitude × 1.8° latitude. It includes a vertically integrated energy–moisture balance atmospheric model, a three-dimensional general circulation model of the ocean and a terrestrial and ocean carbon cycle model^18^. The terrestrial component is a dynamic global vegetation model, including a land surface scheme^52^ based on the Hadley Center model TRIFFID (Top-down Representation of Interactive Foliage and Flora Including Dynamics)^53^. UVic-ESCM has been widely used in a variety of research topics such as climate feedbacks of land cover change^52^,^54^,^55^; climate change and the carbon cycle^8^,^56^,^57^.

UVic-ESCM has also been used for multi-century climate projections in support of the IPCC Fifth Assessment Report^58^. Eby *et al*.^28^ have compared the climate and carbon cycle responses of Earth System Models of Intermediate Complexity (EMICs), including UVic-ESCM, over the historical period against observational estimates. Overall 20th century trends in carbon uptake are reasonably well simulated when compared to observed trends (see Fig. 5 in Eby *et al*.^28^). Weaver *et al*.^18^ showed that present-day atmospheric surface air temperature simulated by UVic-ESCM shows good agreement with annually-averaged NCEP reanalysis climatology^59^, especially on the zonal mean (see Fig. 10 in Weaver *et al*.^18^). However, the UVic-ESCM uses a reduced complexity atmosphere model with no parametrization for clouds and their feedbacks, nor atmospheric transport dynamic processes^18^. Therefore, the UVic-simulated climate effect of irrigation may be less reliable than that of BNU-ESM, and therefore we focus on the effect of irrigation on carbon reservoirs and land surface cover results from UVic-ESCM.

### Simulation experiments

All simulations are forced by the IPCC high-end CO_2_ concentration scenario, RCP8.5^17^. We use the BNU-ESM and UVic-ESCM to conduct idealized desert irrigation geoengineering simulations from year 2020 to 2100, which include simulations of irrigating deserts in Northwest China, Australia and North Africa, individually, and all together (Fig. 2a and Supplementary Table S1). In these simulations, we consider only CO_2_ radiative forcing. We neglect radiative forcing from aerosols and other greenhouse gases, which roughly cancel each other, will have little effect on our simulated impact of irrigation on carbon reservoir and climate change. In each scenario we simulate irrigation by forcing soil moisture to be constant at 360 kg m^−2^ for UVic (following Keller *et al*.^8^) and by daily restoring the moisture content in the 3.433 m deep BNU-ESM soil column to be 800 mm thick, so as to avoid excessive runoff into ocean from the irrigated regions.

### Statistical tests

We use the Student’s t-test since the model output are quite Normal applied to each grid point on the maps in the Figs 2,4 and Supplementary Figs S4–6,S9 and S13. Hatched areas in these figures are regions where changes are not statistically significant at the 5% level. The Null hypotheses are “*GE_Globe* = *GE_none*” over the period of 2071 to 2100 (n = 30) for *GE_Globe* results, “*GE_China* = *GE_none*” for *GE_China* results, “*GE_Australia* = *GE_none*” for *GE_Australia* results and “*GE_Africa* = *GE_none*” for *GE_Africa* results. There are 8192 grid points of temperature, albedo, precipitation and precipitation minus evapotranspiration (P–E) annual mean BNU-ESM data, and 3527 land grid points for BNU-ESM and 2543 land grid points for UVic-ESCM of land carbon, net primary productivity (NPP), soil respiration (RES) and net ecosystem productivity (NEP, NPP minus RES) annual mean data. The returned probability for each grid is two-tailed. The Student’s t-test and plot of these maps in Figs 2,4 and Supplementary Figs S4–6,S9 and S13 were produced using NCAR Command Language (NCL)^60^ version 6.1.2.

## Additional Information

**How to cite this article:** Cheng, W. *et al*. Simulated climate effects of desert irrigation geoengineering. *Sci. Rep.* **7**, 46443; doi: 10.1038/srep46443 (2017).

**Publisher's note:** Springer Nature remains neutral with regard to jurisdictional claims in published maps and institutional affiliations.

## Supplementary Material

Supplementary Information

## Figures and Tables

**Table 1:** Desert irrigation geoengineering areas, water required and induced changes in carbon and climate using BNU-ESM during 2071–2100.

Simulations	Areas (million km^2^)	Water required (Gt)		Δ Total carbon^♯^ (Pg C)		Δ surface albedo^♯^		Δ Surface temperature^♯^ (°C)		Δ Precipitation minus evapotranspiration^♯^ (mm yr^−1^)	
		Initial	Add per year	Irrigated area	Non-irrigated area on land	Irrigated area	Non-irrigated area on land	Irrigated area	Non-irrigated area on land	Irrigated area	Non-irrigated area on land
*GE_Globe*^€^	12.31	2722	3766	104.7	14.6	−0.152	−0.004	−4.20	−0.15	−276	30
*GE_China*^€^	2.33	312	3008	26.1	2.7	−0.091	0	−1.97	−0.06	−298	6
*GE_Australia*^€^	2.28	98	416	0.9	−13.1	−0.029	−0.001	−1.62	−0.01	−207	−8
*GE_Africa*^€^	7.70	2292	633	79.7	12.6	−0.207	−0.003	−5.43	−0.19	−333	42

^€^See Supplementary Table S1, and the irrigated areas have been showing boxed regions in Fig. 2(a).

^♯^Results are 30 years global mean anomalies relative to *GE_none*.
