# Supplementary material for: Simulated climate effects of desert irrigation geoengineering
Source: Sci Rep. 2017 Apr 18;7:46443. doi: 10.1038/srep46443 (PMC5394461; doi:10.1038/srep46443)
Supplement: Supplementary Information [file srep46443-s1.doc]

**Supplementary Information**

**Simulated climate effects of desert irrigation geoengineering**

Wei Cheng^1^, John C. Moore^1,*^, Long Cao^2^, Duoying Ji^1^ and Liyun Zhao^1^

^1^Joint Center for Global Change Studies, College of Global Change and Earth System Science, Beijing Normal University, Beijing 100875, China,

^2^School of Earth Science, Zhejiang University, Hangzhou, Zhejiang 310027, China.

^*^Correspondence and requests for materials should be addressed to J.C.M. (email: john.moore.bnu@gmail.com)

This supplementary information file has 2 tables and 13 figures.

**Supplementary Tables**

| Simulations | Descriptions | Periods  (year) | CO_2_ concentration scenarios |
| --- | --- | --- | --- |
| *GE_none* | No Irrigation | 2006-2100 | RCP8.5 concentrations |
| *GE_Globe* | Irrigation of Northwest China, North African, and Australian deserts | 2020-2100 |
| *GE_China* | Irrigation of Northwest China deserts |
| *GE_Australia* | Irrigation of Australian deserts |
| *GE_Africa* | Irrigation of North African deserts |

**Table S1.** Model simulated experiments.

| Property | Periods (year) | *GE_none* | *GE_Globe* | *GE_China* | *GE_Australia* | *GE_Africa* |
| --- | --- | --- | --- | --- | --- | --- |
| Net ecosystem productivity (Pg C yr^-1^) | 2031-2060 | 2.08 | 1.53 | 0.44 | 0.01 | 1.42 |
|  | 2071-2100 | 1.23 | 1.82 | 0.48 | 0.15 | 1.12 |
| Net primary productivity (Pg C yr^-1^) | 2031-2060 | 61.14 | 11.72 | 2.13 | -0.10 | 10.11 |
|  | 2071-2100 | 72.02 | 11.59 | 2.02 | 0.05 | 9.92 |
| Soil respiration (Pg C yr^-1^) | 2031-2060 | 58.15 | 10.20 | 1.69 | 0.10 | 8.69 |
|  | 2071-2100 | 69.84 | 9.73 | 1.52 | -0.08 | 8.78 |
| Vegetation carbon  (Pg C) | 2031-2060 | 978.61 | 11.94 | -2.65 | -1.10 | 12.80 |
|  | 2071-2100 | 1006.88 | 50.90 | 4.94 | -11.68 | 40.81 |
| Soil carbon  (Pg C) | 2031-2060 | 821.68 | 55.57 | 15.55 | -0.67 | 43.63 |
|  | 2071-2100 | 871.11 | 68.37 | 23.90 | -0.46 | 51.48 |
| Land total carbon  (Pg C) | 2031-2060 | 1800.29 | 67.51 | 12.90 | -1.77 | 56.43 |
|  | 2071-2100 | 1877.99 | 119.27 | 28.84 | -12.14 | 92.29 |
| Cloud radiative forcing (W m^-2^) | 2031-2060 | -25.68 | -1.06 | -0.18 | -0.16 | -0.92 |
|  | 2071-2100 | -26.31 | -1.16 | -0.06 | -0.05 | -0.94 |
| Latent heat flux (W m^-2^) | 2031-2060 | 85.85 | 2.77 | 0.33 | 0.11 | 2.12 |
|  | 2071-2100 | 89.63 | 2.42 | 0.18 | 0.08 | 2.11 |
| Surface albedo | 2031-2060 | 0.151 | -0.008 | -0.002 | -0.001 | -0.006 |
|  | 2071-2100 | 0.138 | -0.005 | 0 | -0.001 | -0.004 |
| Surface temperature (°C) | 2031-2060 | 15.87 | 0.22 | 0.07 | -0.01 | 0.08 |
|  | 2071-2100 | 18.07 | -0.04 | -0.05 | -0.02 | -0.05 |
| Surface land temperature (°C) | 2031-2060 | 11.21 | -0.14 | 0.06 | -0.09 | -0.24 |
|  | 2071-2100 | 13.96 | -0.48 | -0.09 | -0.04 | -0.46 |
| Surface ocean temperature (°C) | 2031-2060 | 17.80 | 0.37 | 0.07 | 0.03 | 0.22 |
|  | 2071-2100 | 19.78 | 0.14 | -0.03 | -0.01 | 0.12 |
| Precipitation (mm yr^-1^) | 2031-2060 | 1081.10 | 34.78 | 4.15 | 1.44 | 26.63 |
|  | 2071-2100 | 1128.93 | 30.39 | 2.26 | 1.01 | 26.51 |
| Land precipitation (mm yr^-1^) | 2031-2060 | 869.78 | 103.84 | 10.70 | -3.67 | 88.51 |
|  | 2071-2100 | 920.41 | 99.63 | 11.84 | -3.17 | 101.16 |
| Ocean precipitation (mm yr^-1^) | 2031-2060 | 1168.76 | 6.14 | 1.44 | 3.56 | 0.97 |
|  | 2071-2100 | 1215.42 | 1.66 | -1.71 | 2.74 | -4.46 |
| Evaporation (mm yr^-1^) | 2031-2060 | 1081.03 | 34.80 | 4.11 | 1.44 | 26.67 |
|  | 2071-2100 | 1128.88 | 30.39 | 2.22 | 1.00 | 26.47 |
| Precipitation minus evapotranspiration (mm yr^-1^) | 2031-2060 | 0.075 | -0.016 | 0.043 | -0.002 | -0.035 |
|  | 2071-2100 | 0.043 | 0.001 | 0.041 | 0.012 | 0.037 |
| Antarctic Circumpolar Current (Sv) | 2031-2060 | 160.74 | 4.02 | -0.49 | 2.34 | 1.30 |
|  | 2071-2100 | 174.15 | 2.59 | 2.14 | -0.33 | 2.57 |
| Atlantic Meridional Overturning Circulation (Sv) | 2031-2060 | 25.56 | 0.13 | 0.59 | 0.34 | -0.31 |
|  | 2071-2100 | 22.75 | -0.82 | -0.14 | 0.32 | -1.46 |

**Table S2.** Desert irrigation geoengineering induced changes in carbon, climate and key Earth system properties. See Supplementary Table S1, and the irrigated areas have been showing boxed regions in figure 2 (a). All results are 30 years global mean values and anomalies are relative to *GE_none* using BNU-ESM*.*

**Supplementary Figures**

**
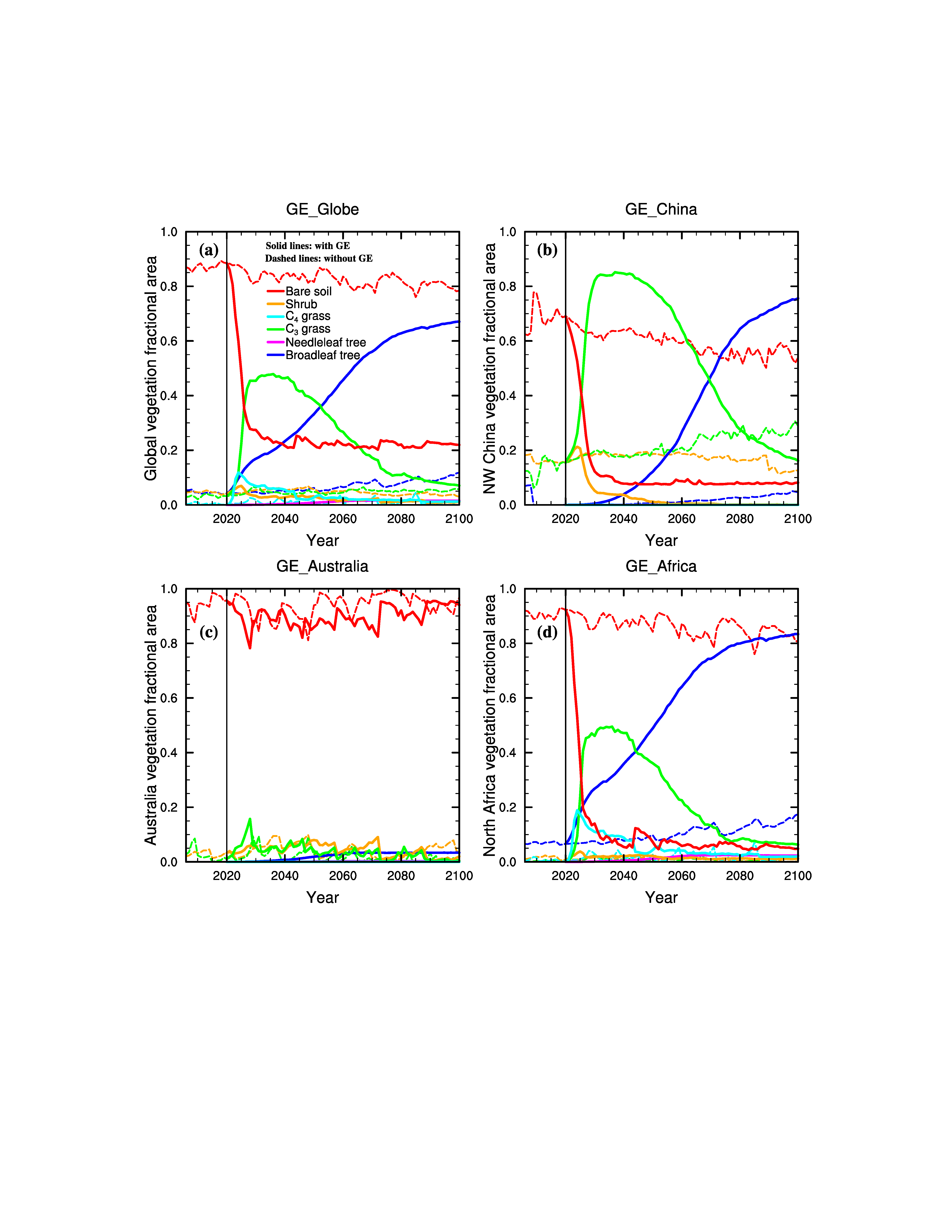
**

**Figure S1.** BNU-ESM simulated vegetation area fraction changes in specific regions and irrigation scenarios. Irrigation is assumed to start at 2020 (solid lines), *GE_none* is shown as dashed lines. This figure was plotted using NCAR Command Language (NCL)^S1^ version 6.1.2 (http://www.ncl.ucar.edu/).


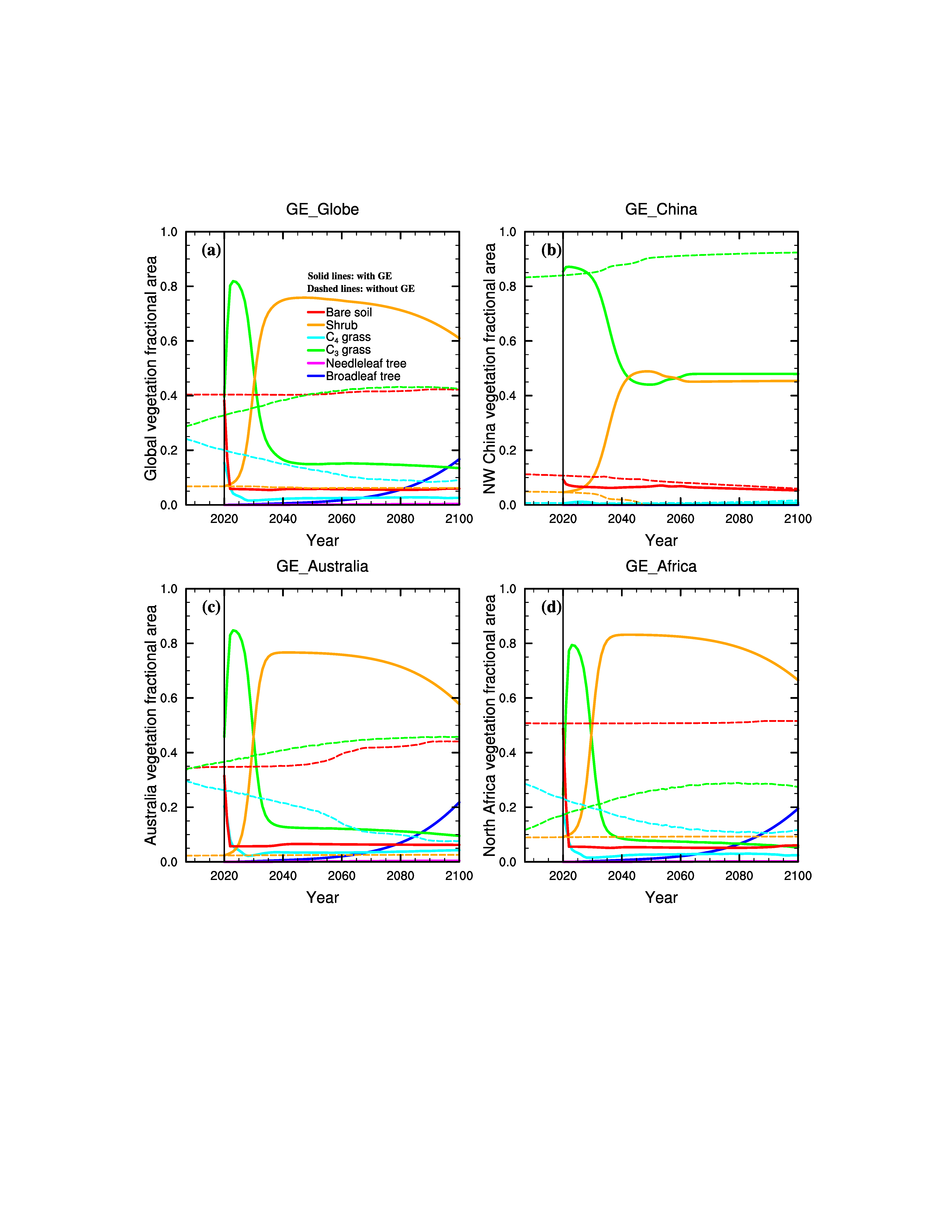


**Figure S2.** As Figure S1 but for UVic-ESCM. Northwest China is simulated to be arid grassland rather than a desert by bare soil fraction in the UVic ESCM. This figure was plotted using NCAR Command Language (NCL)^S1^ version 6.1.2 (http://www.ncl.ucar.edu/).


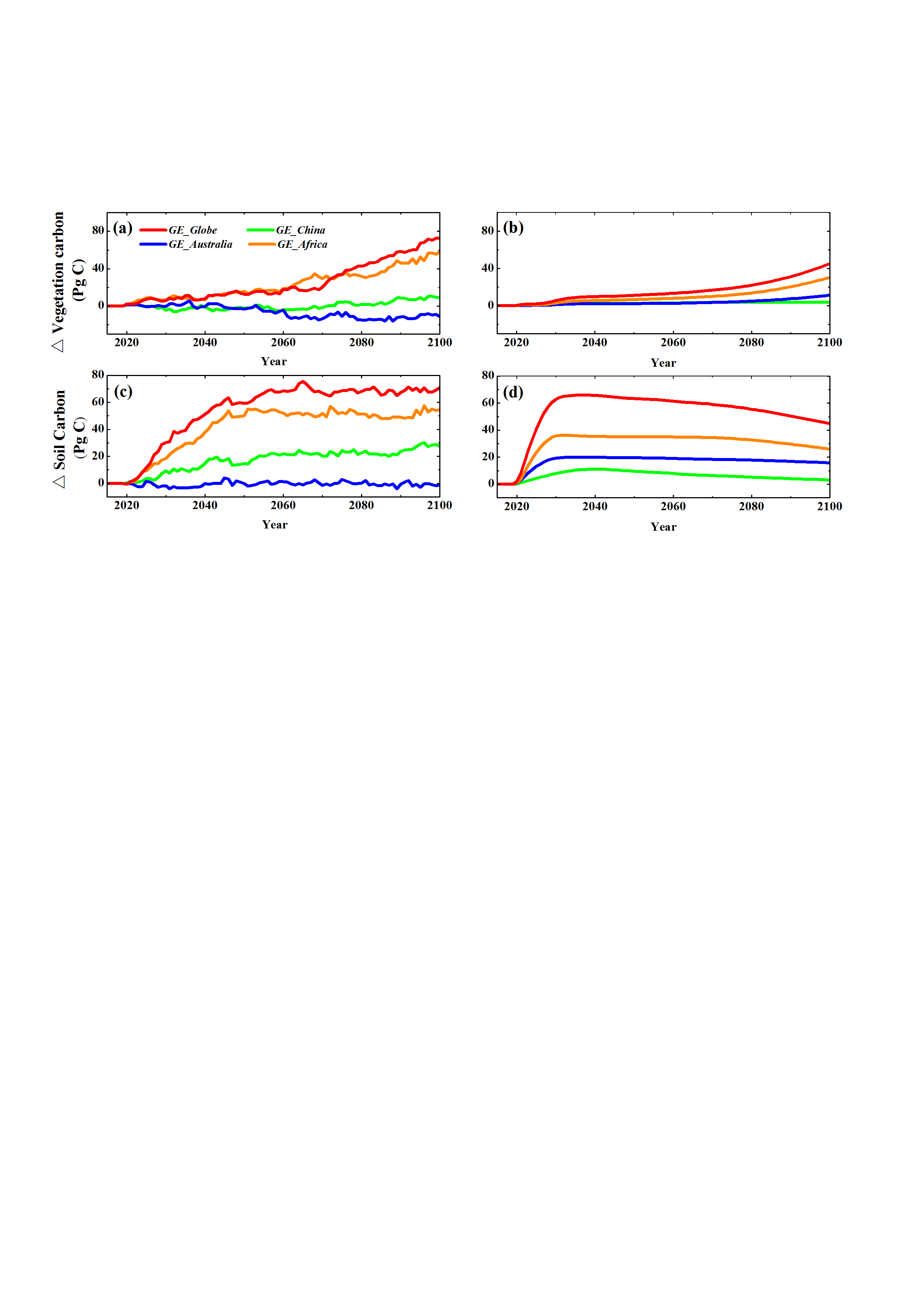


**Figure S3.** Simulated temporal evolution of carbon changes anomalies, relative to *GE_none,* due to irrigation desert geoengineering. BNU-ESM results (a, c) are shown in the left column, and UVic-ESCM results (b, d) are shown in the right column. (a, b) global vegetation carbon; (c, d) global soil carbon. Desert irrigation starts from 2020 to 2100. Values are annual global means. This figure was plotted using Origin version 8.5 from OriginLab.


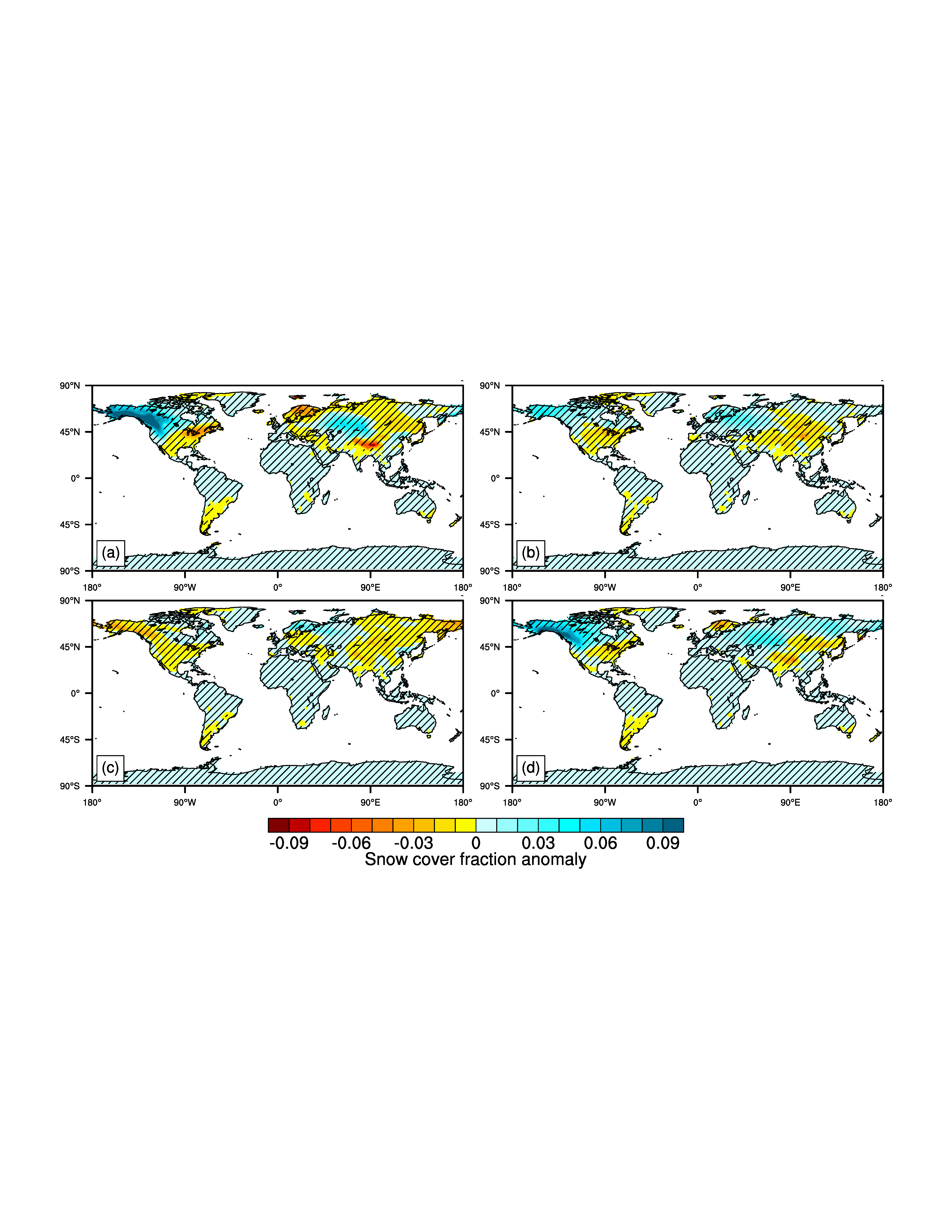


**Figure S4.** BNU-ESM simulated snow cover fraction anomalies for desert irrigation compared with *GE_none* for 2071-2100. (a) *GE_Globe*, (b) *GE_China*, (c) *GE_Australia* and (d) *GE_Africa*. Hatched areas are regions where changes are not statistically significant at the 5% level using the Student’s t-test. The Student’s t-test and maps were produced using NCAR Command Language (NCL)^S1^ version 6.1.2 (http://www.ncl.ucar.edu/).


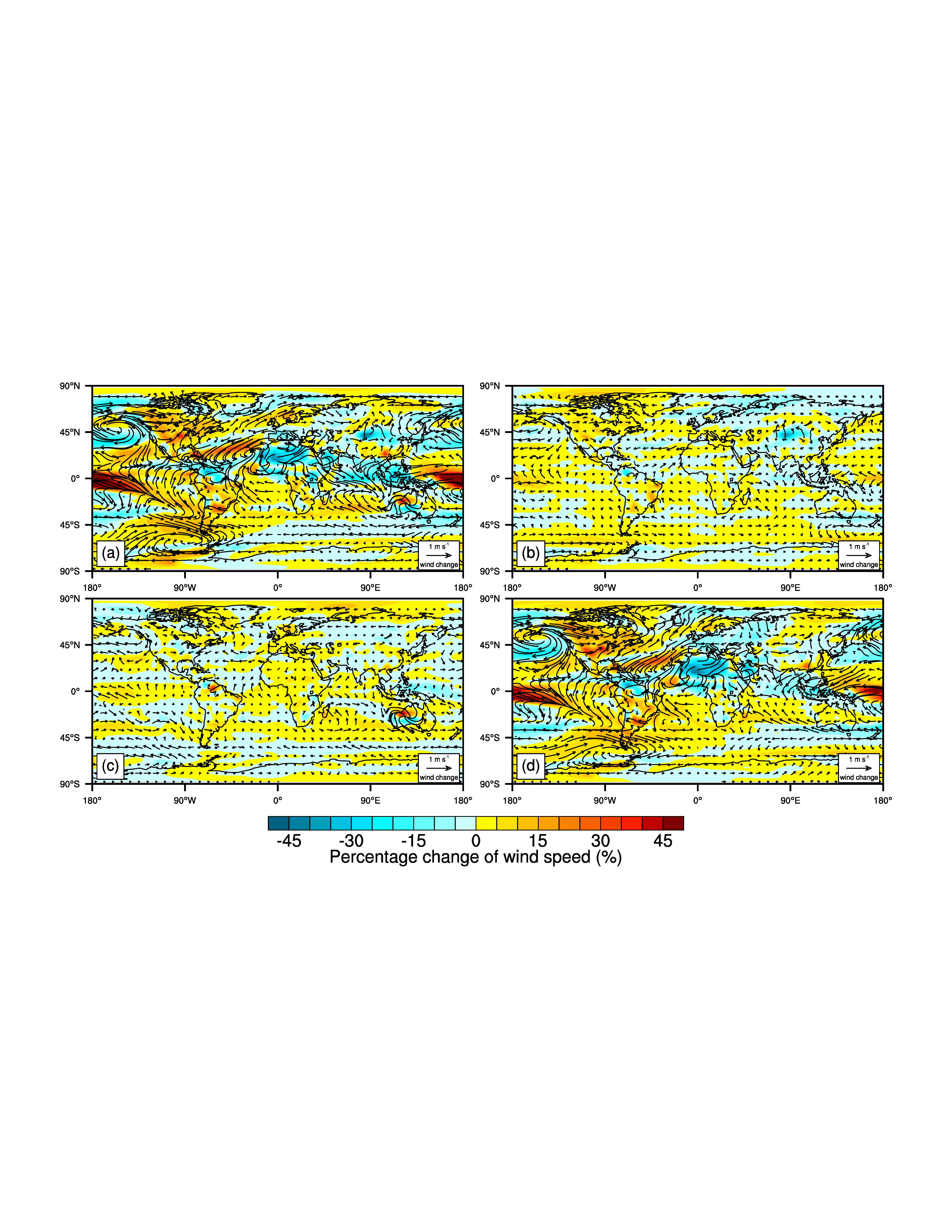


**Figure S5.** BNU-ESM simulated surface wind changes (vector) and the percentage change of wind speed (contours, contour interval is 5%) for desert irrigation compared with *GE_none* for 2071-2100. (a) *GE_Globe*, (b) *GE_China*, (c) *GE_Australia* and (d) *GE_Africa*. The maps were produced using NCAR Command Language (NCL)^S1^ version 6.1.2 (http://www.ncl.ucar.edu/).


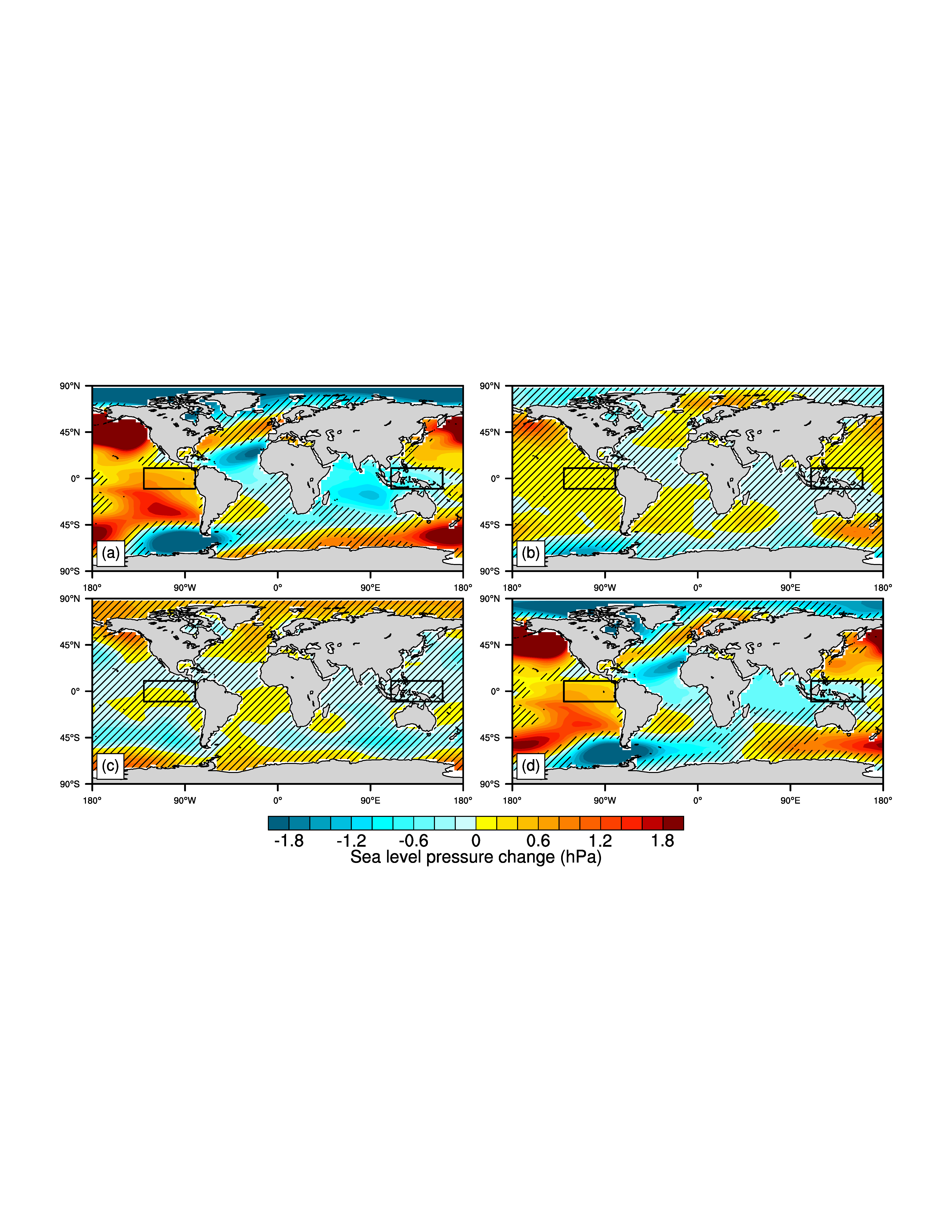


**Figure S6.** BNU-ESM simulated sea level pressure changes for desert irrigation compared with *GE_none* for 2071-2100. (a) *GE_Globe*, (b) *GE_China*, (c) *GE_Australia* and (d) *GE_Africa*. The boxed regions in (a) show where the average sea level pressure was calculated in Fig. S11. Hatched areas are regions where changes are not statistically significant at the 5% level using the Student’s t-test. The Student’s t-test and maps were produced using NCAR Command Language (NCL)^S1^ version 6.1.2 (http://www.ncl.ucar.edu/).

**
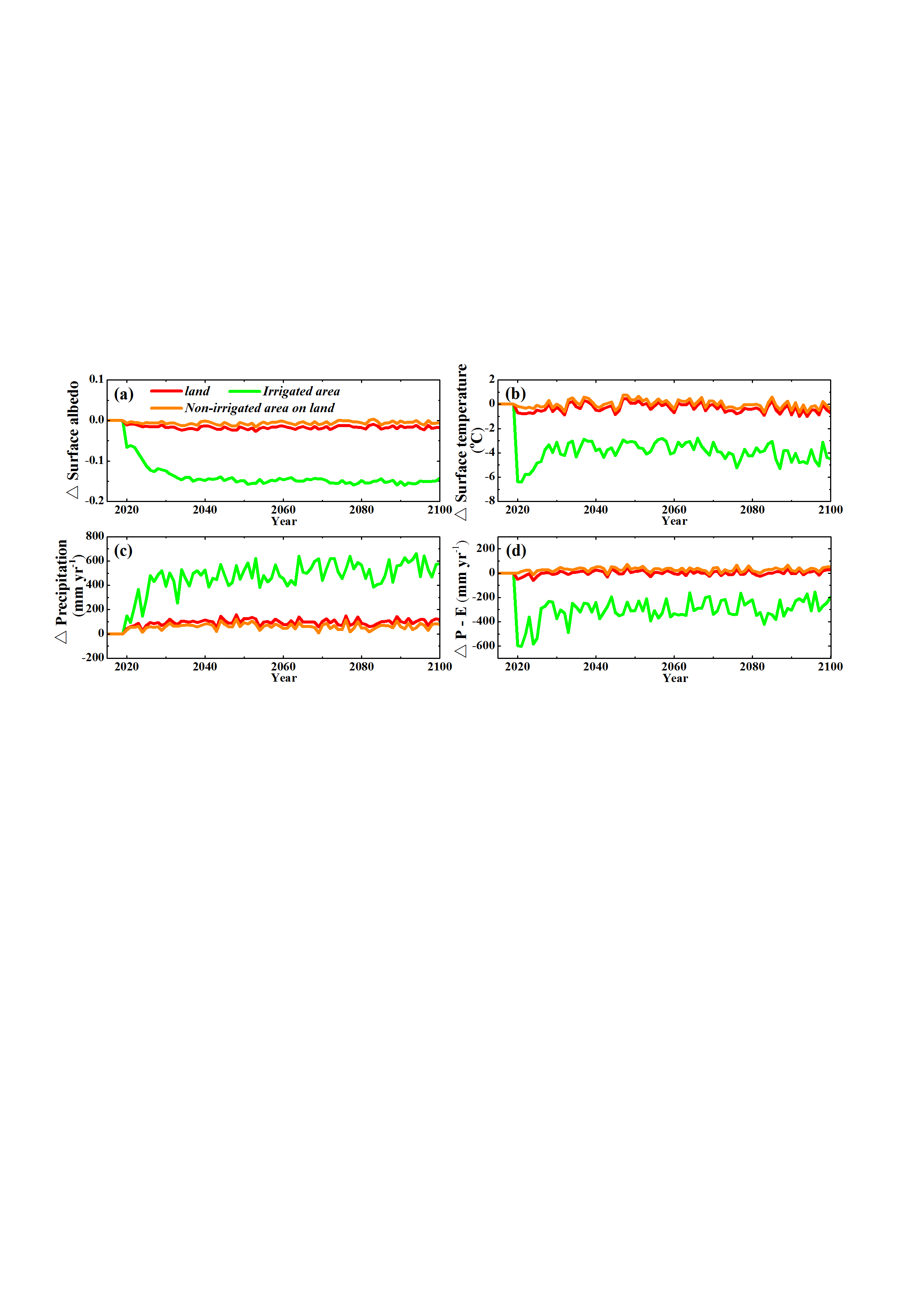
**

**Figure S7.** BNU-ESM simulated temporal evolution of climate changes anomalies, relative to *GE_none,* due to irrigation global desert geoengineering (*GE_Globe*) for irrigated and non-irrigated regions. (a) surface albedo; (b) surface air temperature; (c) precipitation and (d) precipitation minus evapotranspiration (P-E). Desert irrigation starts from 2020 to 2100. Values are annual global means. This figure was plotted using Origin version 8.5 from OriginLab.


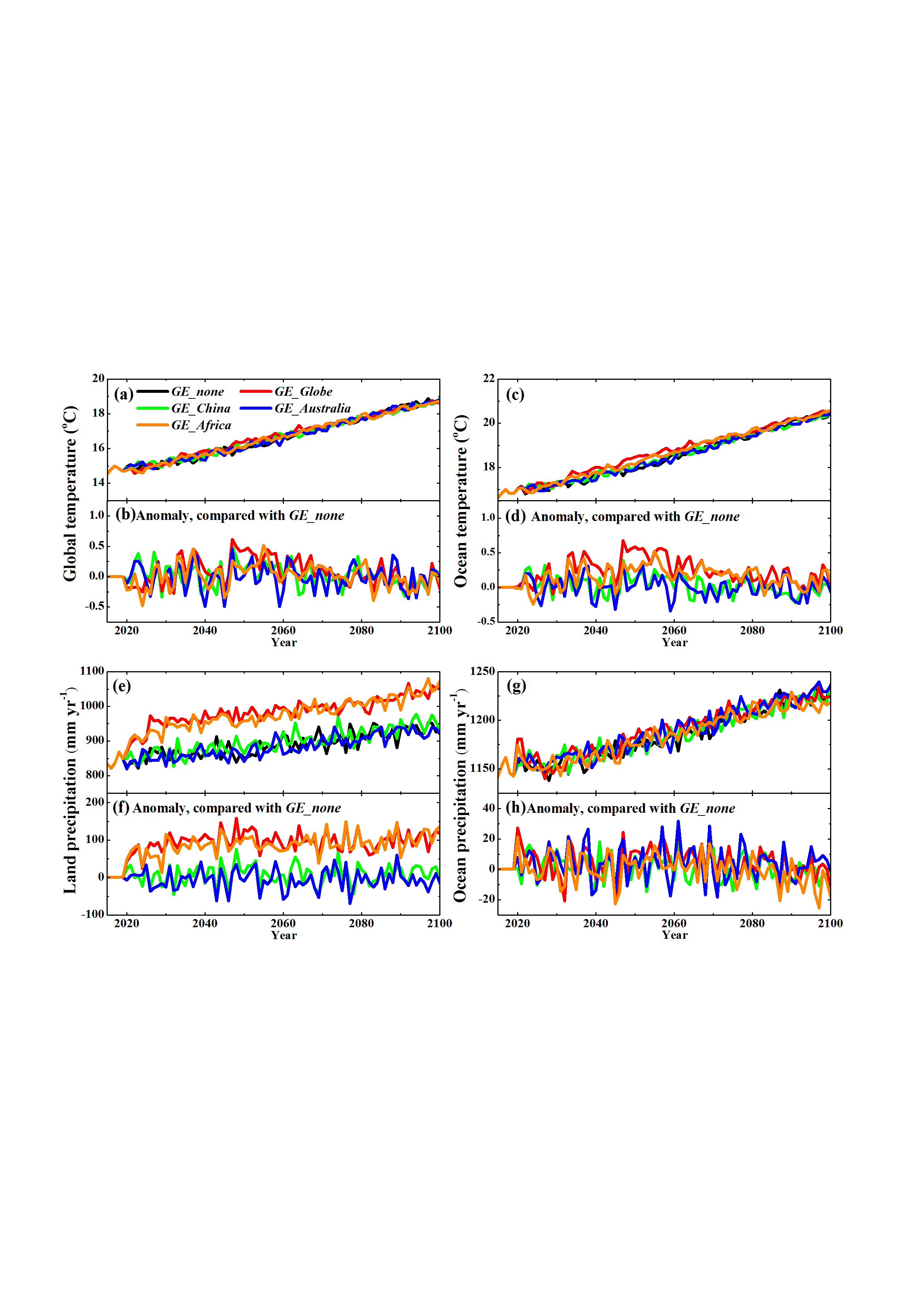


**Figure S8.** BNU-ESM simulated temporal evolution of climate changes in the simulations and anomalies, relative to *GE_none,* due to irrigation desert geoengineering. (a, b) global average surface air temperature; (c, d) global average ocean surface air temperature; (e, f) global average land precipitation and (g, h) global average ocean precipitation. Desert irrigation starts from 2020 to 2100. Values are annual global means. This figure was plotted using Origin version 8.5 from OriginLab.


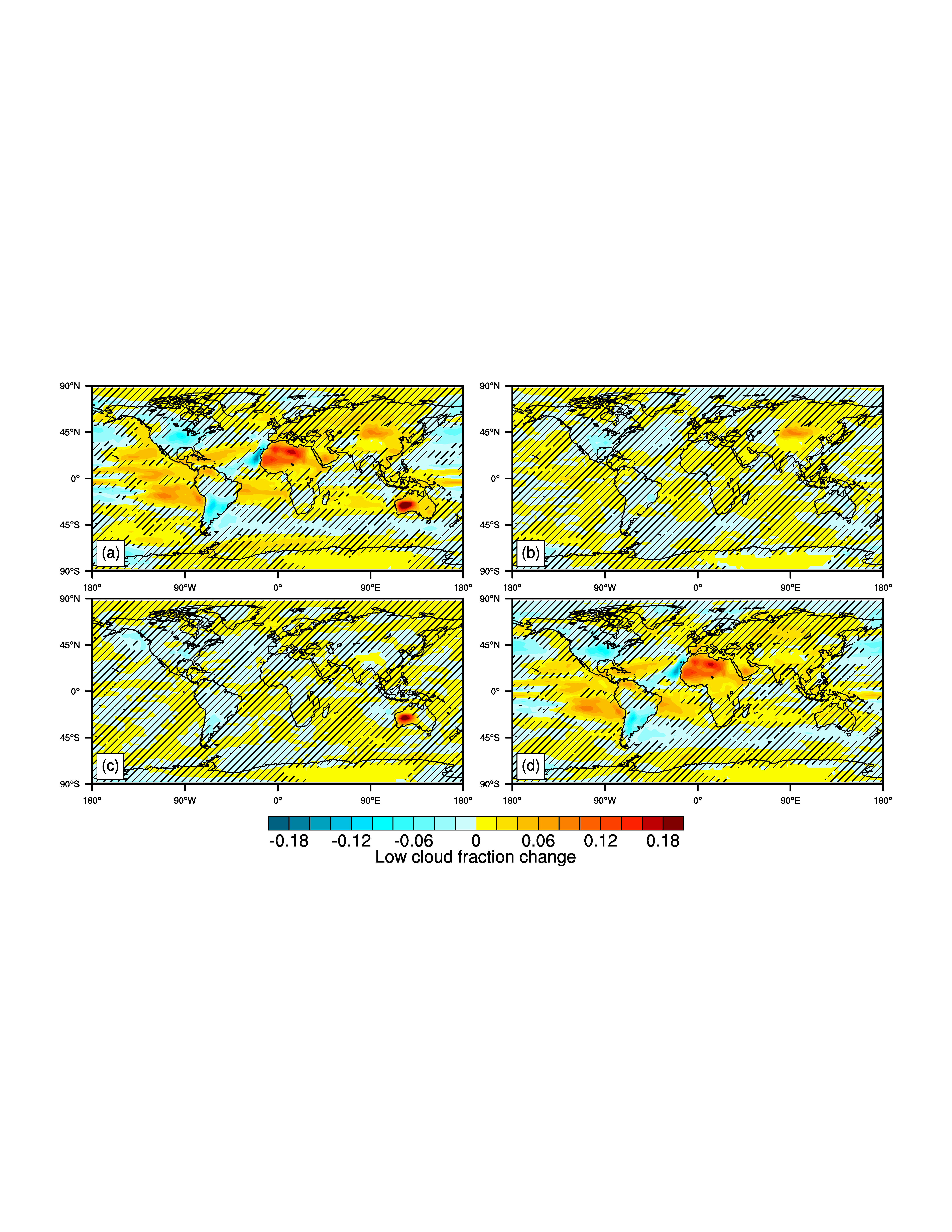


**Figure S9.** BNU-ESM simulated change in low cloud cover for desert irrigation compared with *GE_none* for 2071-2100. (a) *GE_Globe*, (b) *GE_China*, (c) *GE_Australia* and (d) *GE_Africa*. Hatched areas are regions where changes are not statistically significant at the 5% level using the Student’s t-test. The Student’s t-test and maps were produced using NCAR Command Language (NCL)^S1^ version 6.1.2 (http://www.ncl.ucar.edu/).


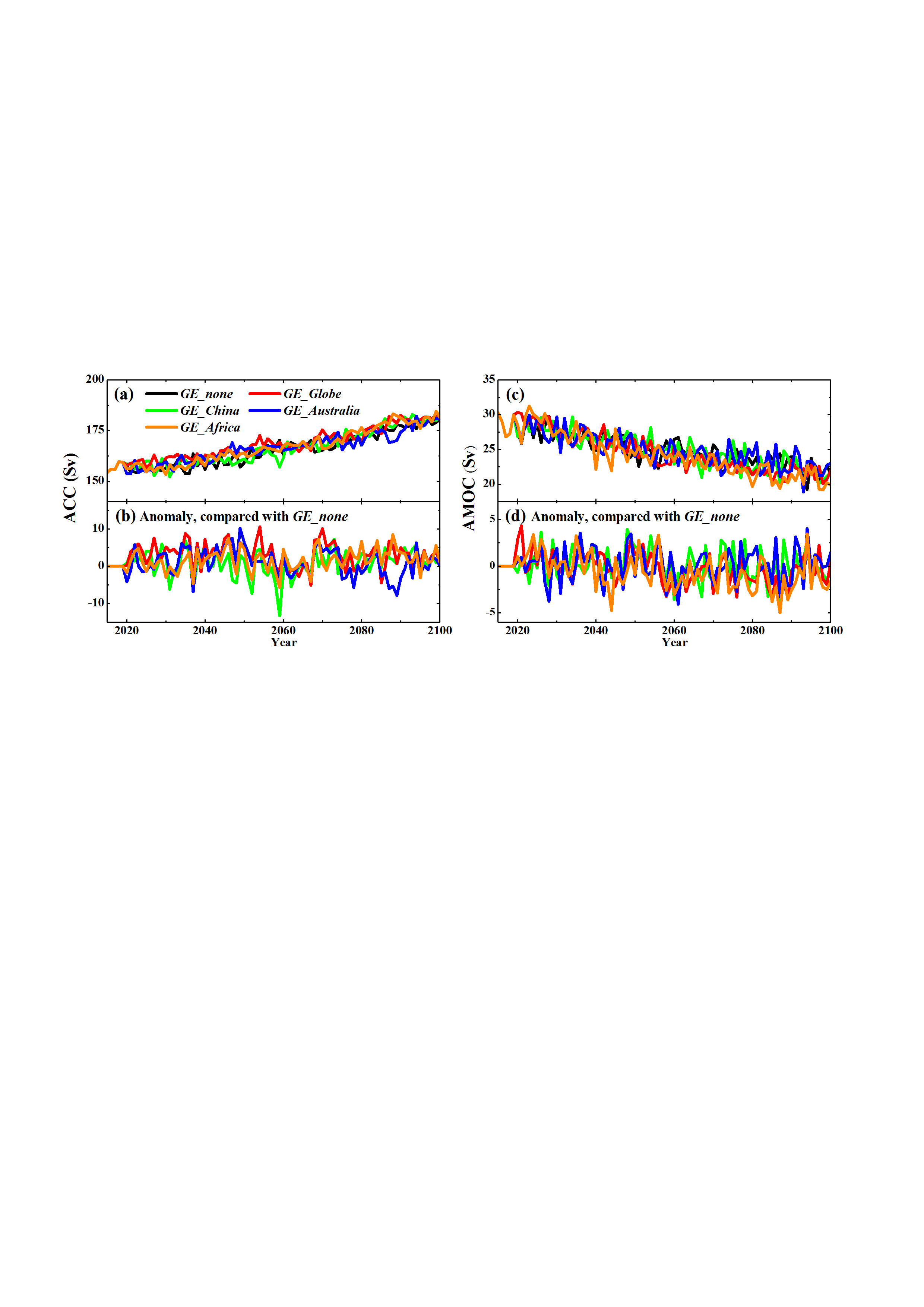


**Figure S10.** BNU-ESM simulated temporal evolution of ocean circulation changes in the simulations and anomalies, relative to *GE_none,* due to irrigation desert geoengineering. (a, b) ACC (Antarctic Circumpolar Current); (c, d) AMOC (Atlantic Meridional Overturning Circulation). Desert irrigation starts from 2020 to 2100. Values are annual global means. This figure was plotted using Origin version 8.5 from OriginLab.


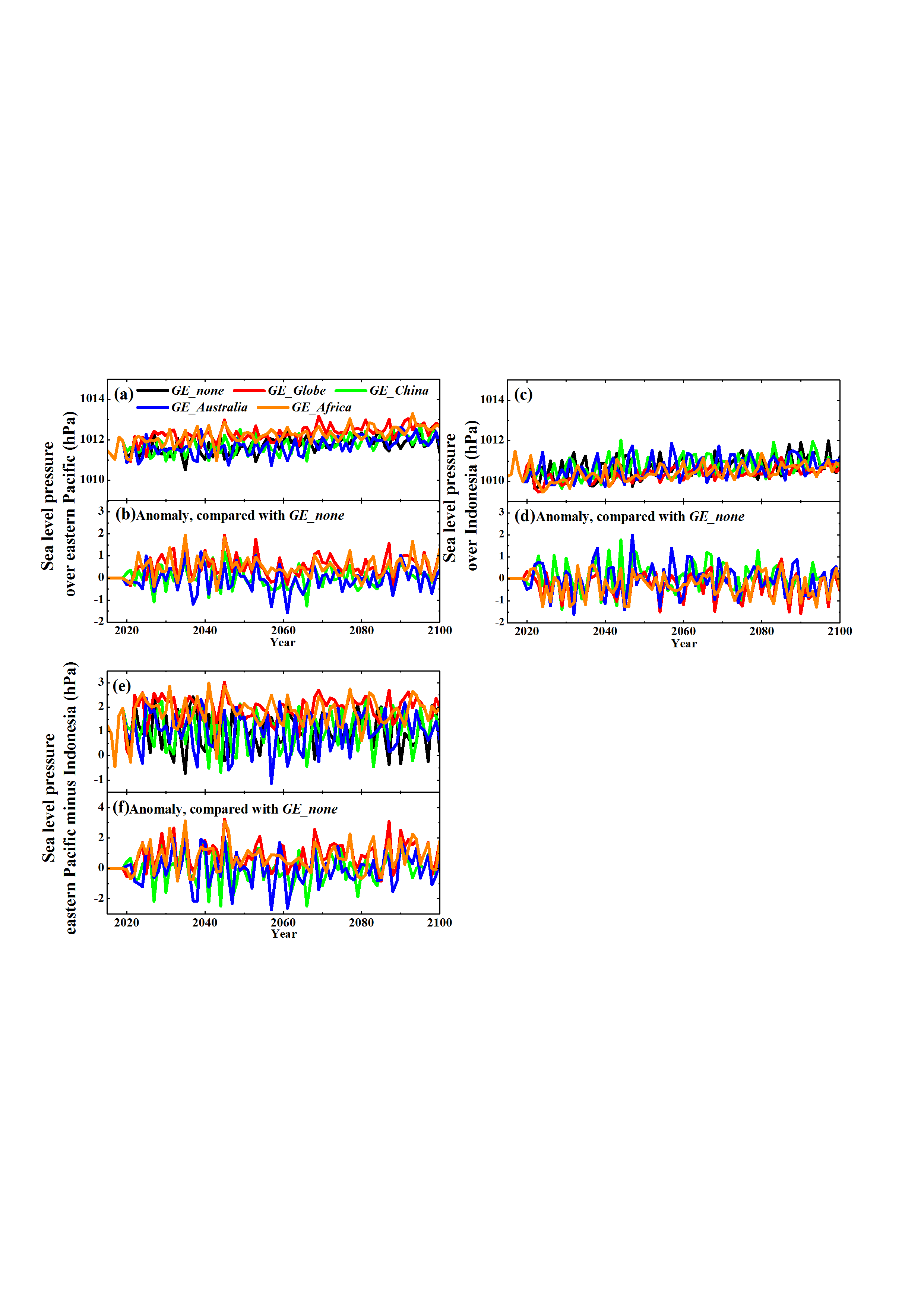


**Figure S11.** BNU-ESM simulated temporal evolution of sea level pressure changes in the simulations and anomalies, relative to *GE_none,* due to irrigation desert geoengineering. (a, b) sea level pressure treads for the region over eastern Pacific Ocean (130^o^-80^o^ W, 10^o^ S-10^o^ N); (c, d) sea level pressure treads for the region over Indonesia (110^o^-160^o^ E, 10^o^ S-10^o^ N); (e, f) sea level pressure trends difference between the region over eastern Pacific Ocean and Indonesia. The boxed regions in Fig. S6 (a) show where the average sea level pressure was calculated. Desert irrigation starts from 2020 to 2100. Values are annual global means. This figure was plotted using Origin version 8.5 from OriginLab.


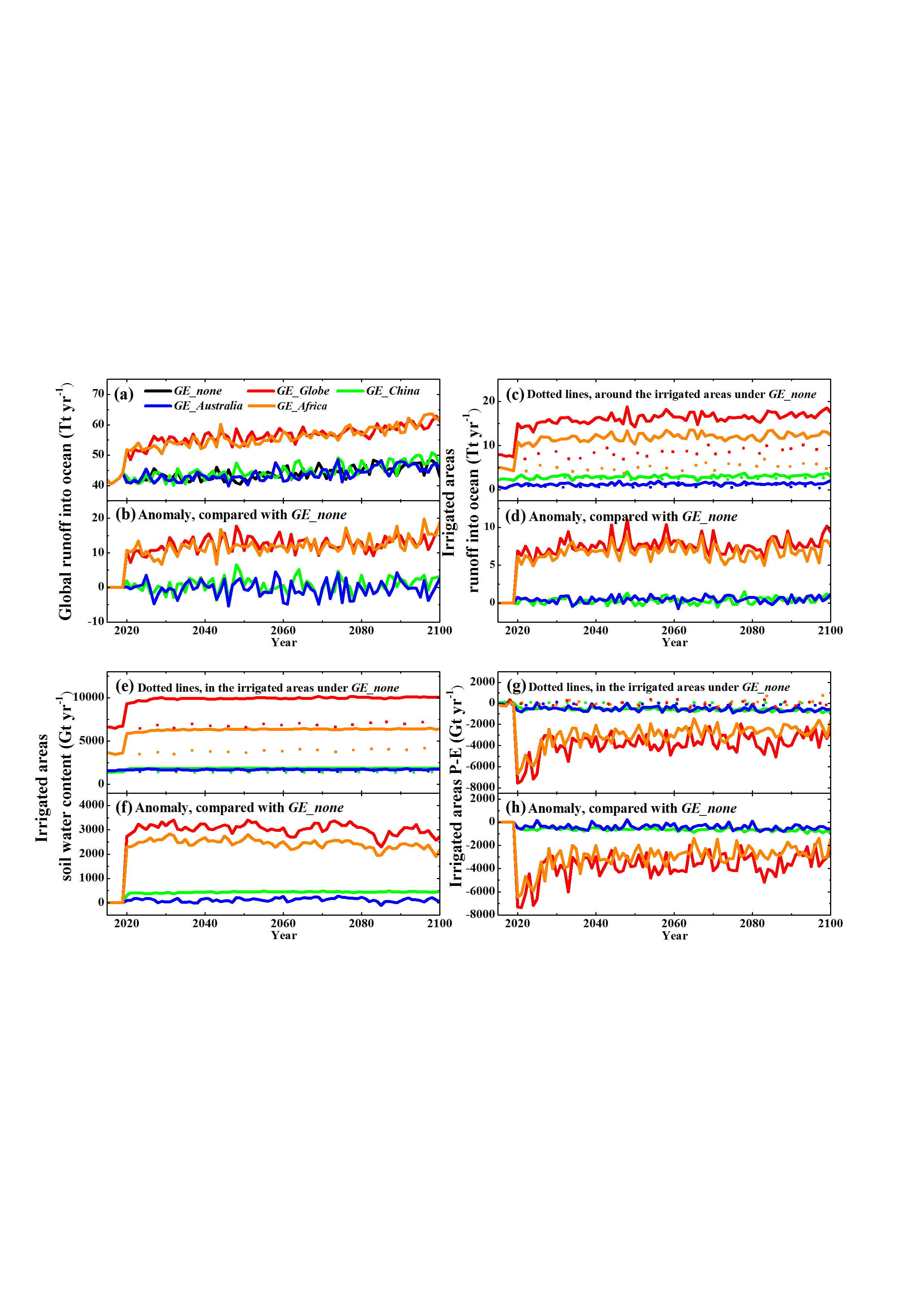


**Figure S12.** BNU-ESM simulated temporal evolution of water changes in the simulations and anomalies, relative to *GE_none,* due to irrigation desert geoengineering. (a, b) global runoff discharge into ocean; (c, d) runoff discharge into ocean around the irrigated areas; (e, f) soil water content in the irrigated areas and (g, h) precipitation minus evaporation (P-E) in the irrigated areas. Desert irrigation starts from 2020 to 2100. Values are annual global means. This figure was plotted using Origin version 8.5 from OriginLab.


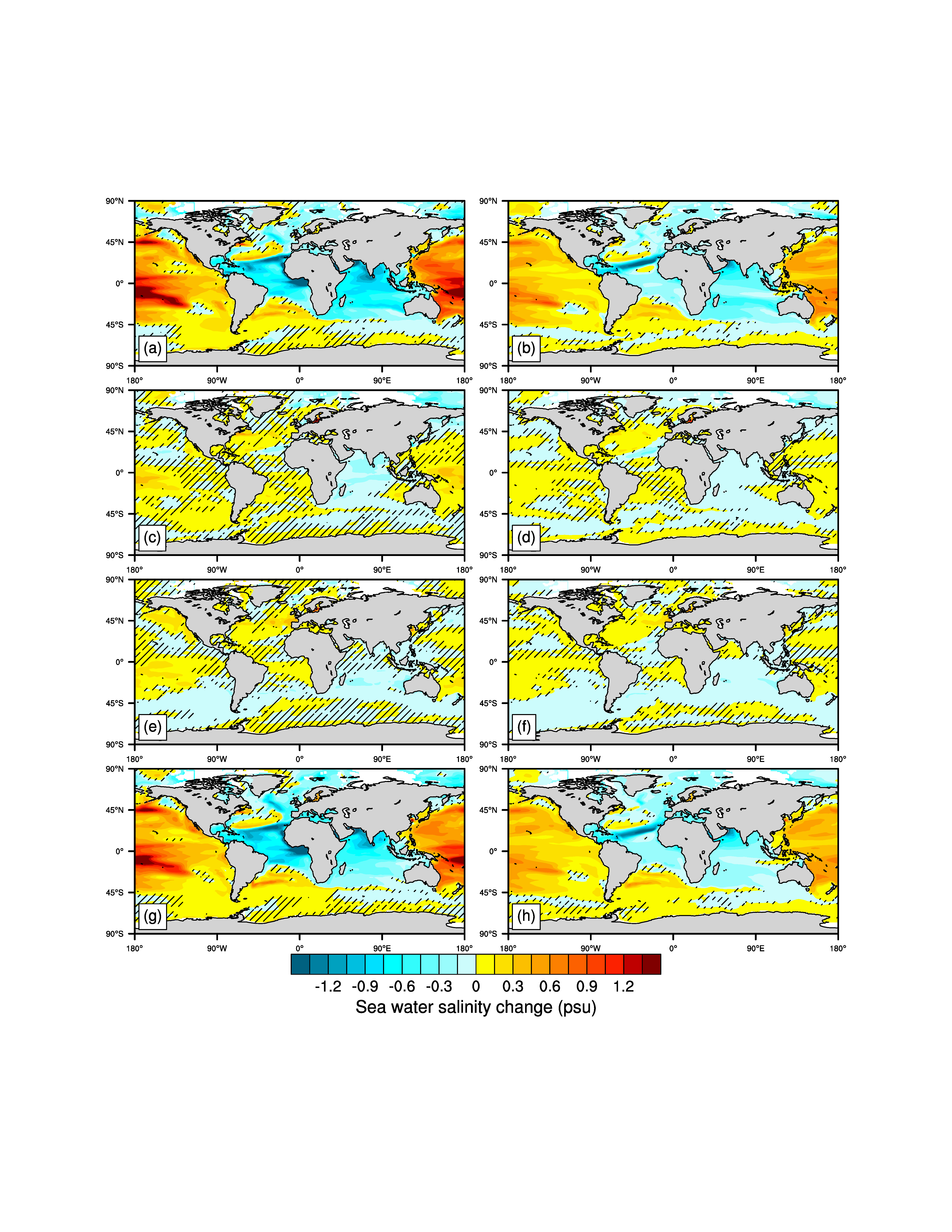


**Figure S13.** BNU-ESM simulated sea water salinity anomalies of desert irrigation compared with *GE_none* over the period of 2071 to 2100. Surface salinity change results are shown in the left column, and average salinity change in upper 302 m of ocean results are shown in the right column. (a, b) *GE_Globe*; (c, d) *GE_China*; (e, f) *GE_Australia*; (g, h) *GE_Africa*. Hatched areas are regions where changes are not statistically significant at the 5% level using the Student’s t-test. The Student’s t-test and maps were produced using NCAR Command Language (NCL)^S1^ version 6.1.2 (http://www.ncl.ucar.edu/).

**Supplementary Reference**

S1. Boulder, Colorado: UCAR/NCAR/CISL/TDD. The NCAR Command Language (Version 6.1.2) [Software]. http://dx.doi.org/10.5065/D6WD3XH5 (2015).
